# Efficacy of knee osteoarthritis by use of laser acupuncture: A systematic review and meta-analysis

**DOI:** 10.1097/MD.0000000000038325

**Published:** 2024-06-21

**Authors:** Rong Han, Chunxia Guo, Kit Lau, Jinlian Hu

**Affiliations:** a Department of Biomedical Engineering, City University of Hong Kong, Kowloon Tong, Hong Kong, China.

**Keywords:** efficacy, laser acupuncture, meta-analysis, osteoarthritis

## Abstract

**Background:**

Previous studies need to be aggregated and updated. We aim to assess the efficacy of laser acupuncture (LA) in knee osteoarthritis (OA) through a meta-analysis.

**Methods:**

Electronic databases were searched for studies investigating laser acupuncture’s efficacy in managing OA. Data were collected from the beginning of each database to 2022 (up to March). The “WOMAC total score,” “WOMAC stiffness score,” “WOMAC pain score,” “WOMAC physical function score,” and “VAS score” were the key outcomes of interest. The Der Simonian-Laird method for random effects was used.

**Results:**

Twenty-five randomized controlled clinical trials met our criteria and were included (2075 patients). Comparisons of interest is the LA versus Sham LA (efficacy), LA versus. A (Acupuncture) (comparative effectiveness), LA combined with A versus A (effectiveness as an adjunct), and any other research used LA in their treatment. Laser irradiation is effective in patients with Knee OA. LA is also effective and has almost the same outcome as laser irradiation. LA can achieve almost the same effect as manual acupuncture, even better than acupuncture in some studies.

**Conclusion:**

Laser acupuncture is more or less effective in patients with OA; better efficacy will be achieved under appropriate laser parameters (810 nm, 785 nm) in the LA versus Sham LA group. Many studies have diverse results, possibly due to unstaged analysis of patients’ disease, inappropriate selection of acupoints, lack of remote combined acupoints, and unreasonable laser parameters. Furthermore, a combination of acupoints was found to be more effective, which aligns with the combined-acupoints application of traditional Chinese medicine.

**Key points**

- Using a meta-analysis to determine the effectiveness of laser acupuncture (LA) in treating osteoarthritis (OA).
- In several trials, LA has been shown to outperform acupuncture in terms of effectiveness when compared to manual acupuncture.
- The results indicated that laser acupuncture is somewhat beneficial for OA patients; optimal results will be obtained with suitable laser parameters (810 nm, 785 nm) on the LA group as opposed to the sham LA group.
- Our results indicate that combining acupoints was found to be more beneficial, which is consistent with traditional Chinese medicine’s use of combining acupoints.

## 1. Introduction

Knee Osteoarthritis (KOA) is a debilitating condition affecting knee joints, leading to cartilage and bone damage and potential disability.^[1]^ Joint discomfort, stiffness, edema, and decreased range of motion, and even weakness or numbness are typical symptoms, significantly impacting daily activities.^[1]^ In an aging society, KOA is prevalent among older individuals, as well as among workers and athletes.^[1,2]^ While acetaminophen and NSAIDs are commonly used for pain management, their long-term use is limited due to adverse effects on the gastrointestinal, renal, cardiac, and hematological systems.^[3]^ Studies suggest that opioids do not offer significant benefits over NSAIDs for OA pain relief and may pose more risks than benefits, prompting interest in complementary medicine.^[2,3]^ The Western Ontario and McMaster University Osteoarthritis Index (WOMAC) plays a crucial role in assessing OA patients’ symptoms and improvement rates, focusing on pain, stiffness, and physical function.^[4]^

Effective treatment is a big challenge for patients with KOA.^[4–6]^ Acupoints are unique in traditional Chinese medicine, mostly in places with many nerve endings and blood vessels.^[7]^ In order to promote traditional Chinese medicine (TCM) theory, several authors interpret distinctions between acupoints or between acupoints and sham points as acupoint specificity.^[7–9]^ Currently, researchers have found mast cells are abundant at acupoint sites.^[9]^ Through the stimulation of certain acupoints along the meridians (channels of acupoints), acupuncture, a traditional Chinese medical treatment, aims to promote health by managing basal energy, which can regulate visceral feeling and function.^[10]^ A crucial component of traditional Chinese treatment is acupuncture, while laser is spread by Western medicine.^[4–6]^ LA is an attractive treatment combining acupuncture and laser to integrate traditional Chinese medicine and Western medicine, being a bridge and a general trend.^[4–6]^ Currently, they cannot replace mainstream medicine but spread to use worldwide.^[4–6]^ LA could achieve short-cycle treatment with better pain relief.^[4,6]^ Apart from pain relief and treatment cycle reduction, other aspects such as psychological well-being and health-being cannot be ignored, which is also a future work for discovering.^[11]^

Compared with traditional acupuncture and moxibustion, the laser acupuncture point therapy instrument has painless, sterile, harmless, simple, and safe advantages.^[6]^ LA is a novel method of acupoint stimulation.^[4–6]^ At the World Association for Photobiomodulation Therapy conference in 2018, Praveen Arany and colleagues gave the first precise definition of LA.^[5]^ It is described as “Initiating therapeutic effects akin to those of needle acupuncture and associated therapies by photonic stimulation of acupuncture sites and locations, in addition to the advantages of photobiomodulation.”^[5]^ Commonly used instruments for acupoint therapy include He-Ne laser therapy devices, carbon dioxide laser therapy, krypton laser therapy, argon laser therapy, and neodymium-doped yttrium aluminum garnet laser therapy devices.^[6]^

Due to the small sample size of trials and diverse laser parameters, meta-analysis is necessary to examine the pooled data for statistical significance.^[12]^ Chen et al have done a meta-analysis on LA treatment on KOA in 2019.^[13]^ But previous meta-analyses need to be updated, and more comparison groups need to be assessed. We searched and obtained clinical trials of LA for treating patients with knee OA, which aims to systematically evaluate the efficacy and safety of LA in treating OA to provide robust evidence support for future clinical large-scale applications.

## 2. Methods

**Figure 1.**
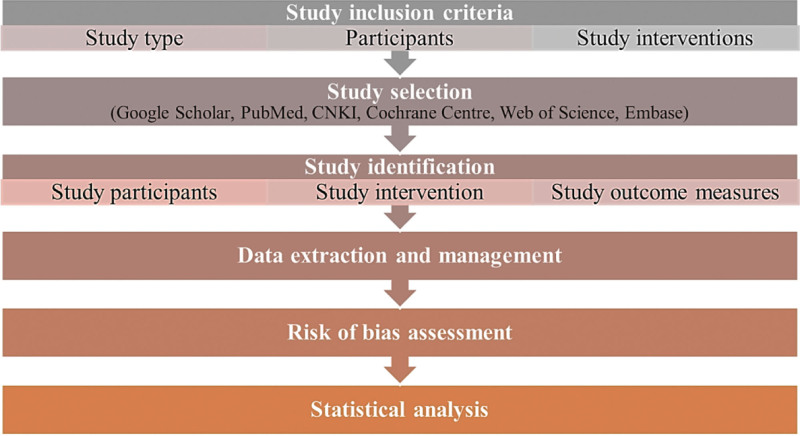
The procedures of meta-analysis.

The meta-analysis has been registered in PROSPERO with ID of CRD42022354798 with the link of https://www.crd.york.ac.uk/prospero/display_record.php?ID=CRD42022354798. The procedures performed in this systematic review and meta-analysis follow guidelines for reporting meta-analysis (PRISMA guidelines).^[12]^ A flow diagram of the procedures is displayed in Figure 1.

### 2.1. Study inclusion criteria

*Study type*: We included all Clinical Controlled Trials published in either English or Chinese. Non-CT, quasi-randomized controlled trial (RCT), retrospective studies, medical record reports, reviews, and summaries of articles were excluded.

*Participants*: Every study participant had a KOA diagnosis. Age, gender, or race restrictions did not apply.

*Study interventions*: Studies of the following interventions of LA. Critical comparisons of interest are LA versus sham LA (efficacy), LA versus acupuncture (Comparative effectiveness), LA combined with A versus the same LA (Effectiveness as an adjunct), multiple sources of LA versus a single source of LA, and any other used LA in their treatment.

### 2.2. Electronic search methods for study identification

Google Scholar, PubMed, CNKI, Cochrane Centre, Web of Science, Embase were the databases searched with the search terms of (“laser acupuncture” OR “laser therapy” OR “photobiomodulation” OR “acupoint laser” or “photo acupuncture”) AND (“osteoarthritis” OR “arthritis” OR “Knee Osteoarthritis” OR “Osteoarthritis of Knee”) AND (“Controlled clinical trial” OR “Clinical trial” OR “Randomized controlled trial” OR “Double-blind method” OR “single-blind method”).

#### 2.2.1. Study selection

**Figure 2.**
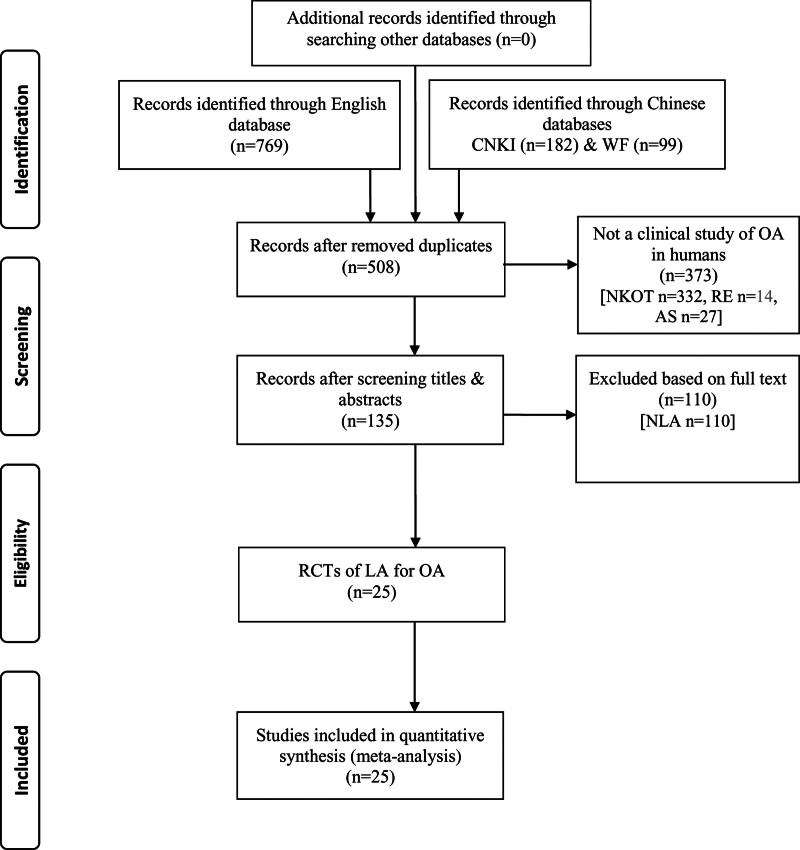
Flow diagram of the search and selection process of CTs of LA for osteoarthritis (OA).

Through the use of electronic searches, 769 records were found; 261 duplicates were eliminated. After examining the full-text publications, 110 studies were eliminated, out of the 373 that were initially eliminated based on their titles and abstracts. Twenty-five CTs in all (2075 patients) fulfilled the requirements for inclusion. Figure 2 shows a thorough flow diagram of the research selection.

#### 2.2.2. Study description

Of the 25 included RCTs, most trials were performed in China^[14–31]^; others were performed in Egypt,^[32]^ Indonesia,^[33]^ UK,^[34]^ Australia,^[35]^ Iran,^[36]^ and Turkey.^[37]^ Three studies own multiple trial centers,^[20,26,30]^ while others have 1 trial center. Fourteen trials used double-blind when conducting experiment,^[14,16,21,26–31,33–35,37,38]^ 3 trials applied single-blind method,^[15,32,36]^ and the others are unclear. Table 1 displays the features of the trials that were included. Besides the detailed parameters of the lasers used are also depicted in the form of Table 1.

**Table 1:** The characteristics of included studies.

Author (year)	Sample size T/C; gender M T/C; age T/C	Study design	Outcome measurements	Laser parameters	Acupoints	Acup-oints no.	Risk of bias	Outcomes description
Nagwa Mohammed, 2018^[32]^	20/20; 55.25 ± 11.373, 54.05 ± 8.420; 3 20, 3 20	LA vs Sham LA	Beta-endorphin Substance P VAS	LW: 808 nm, PD: 2.8 w/cm^2^, OP: 90 mw	*Dubi, Zusanli, Yinlingquan, Xuehai, Yanglingquan*	5	SG: L, AC: U, BPt: L, IOD: L, SOR: L, OB: L	LA exerted a notably improvement in pain on VAS and serum beta-endorphin, a significantly decrease in substance P
Dwi R. Helianthi, 2016^[33]^	20/20; 69 ± 6, 68 ± 5; 12 30, 5 29	LA vs Sham LA	VAS score: 19.7 ± 14.8; 52.86 ± 6 LI: 5.3 ± 4.5; −1.2 ± 3.7	LW: 785 nm, PD: 25 mw/cm^2^, OP: 50 mw	*Dubi, Zusanli, Yinlingquan, Yanglingquan, Neixiyan*	5	SG: L, AC: U, BPt: L, IOD: L, SOR: L, OB: L	LA own Significantly higher VAS scores
A.S. Al Rashoud, 2014^[34]^	26/23; 52 ± 9, 56 ± 11; 10 26, 8 23	LA vs Sham LA	VAS score: 3.4; 5.2 (mean value) SKFS: 26 (14–43), 41 (29–53) AEs: 0/26; 0/23	LW: 830 nm, PD: 4J/cm^2^, OP: 30 mw	-	-	SG: L, AC: U, BPt: L, IOD: L, SOR: L, OB: L	LA own significantly higher VAS scores
Xueyong Shen, 2009^[14]^	19/16; 60.10 ± 6.83, 56.40 ± 7.41; 2 20, 2 20	LA vs Sham LA	WTS: 2.58 ± 1.02; 2.25 ± 1.00 WPS: 7.79 ± 3.42; 6.20 ± 3.68 WSS: 1.73 ± 1.50; 1.63 ± 1.67 WPF: 24.65 ± 14.59; 18.99 ± 11.41 AEs: 3/20; 0/20	LW: 10600 nm; 650 nm, PD: -, OP: 36 mw	*Dubi*	1	SG: L, AC: U, BPt: L, IOD: L, SOR: L, OB: L	The pain reduction of the LA group was 49%, placebo group was 13%
M. Yurtkuran, 2007^[37]^	28/27; 51.83 ± 6.83, 53.478 ± 7.13; 1 28, 1 27	LA vs Sham LA	WTS: 62.53 ± 22.81; 44.63 ± 17.73 WPS: 12.65 ± 5.89; 10.13 ± 4.81 WSS: 5.00 ± 1.87; 3.88 ± 2.03 WPF: 44.88 ± 16.53; 30.63 ± 12.44 NHP: 7.26 ± 5.58, 6.31 ± 5.76 50 foot w: 18.69 ± 2.84, 19.33 ± 4.36 KC: 38.82 ± 3.58, 38.43 ± 3.96 MTS: 3.95 ± 1.65, 4.84 ± 2.03 VAS score: 59.29 ± 2.45; 4.94 ± 2.64	LW: 904 nm, PD: 10 mw/cm^2^, OP: 4 mw	*Yinlingquan*	1	SG: L, AC: L, BPt: L, IOD: L, SOR: L, OB: L	LA own significant improvement in PVAS, 50 foot walk, and KC in group 1. The improvement in KC of LA group was superior to that in placebo group at the 2nd week.
Mei-Kin Li Rees, 2017^[38]^	18/16; 61.3 ± 11.36, 63.0 ± 10.11; 10 20, 10 20	LA vs Sham LA	WTS: 24.48 ± 38.44; 71.38 ± 48.40 WPS: 4.63 ± 8.18; 12.43 ± 10.59 WSS: 3.12 ± 4.35; 6.47 ± 4.80 WPF: 20.64 ± 21.37; 54.05 ± 30.27 VAS score: 0.69 ± 1.17; 3.08 ± 2.5 MSS: 3.95 ± 5.08, 7.50 ± 5.08 MAS: 0.25 ± 0.64, 1.75 ± 1.86 MSA: 4.20 ± 5.47, 9.25 ± 9.24 PPI: 0.65 ± 0.93, 1.80 ± 1.06 CCS: 22.70 ± 4.63, 18.75 ± 6.59 CAS: 22.00 ± 5.04, 15.80 ± 7.23 CTFS: 44.70 ± 9.10, 34.55 ± 13.32 WAIT: 26.05 ± 2.48, 22.65 ± 4.56 WAIBS: 26.55 ± 2.63, 23.60 ± 4.17 WAIG: 18.50 ± 3.69, 17.25 ± 3.65 WAIA: 71.10 ± 7.05, 63.50 ± 9.93 MHLCIB: 24.20 ± 8.10, 23.65 ± 4.53 MHLCC: 16.70 ± 7.94, 15.10 ± 8.53 MHLCPO: 23.55 ± 6.33, 22.10 ± 4.44 MHLCDS: 13.80 ± 3.33, 13.60 ± 2.56 MHLCOPS: 9.75 ± 4.19, 8.50 ± 3.28 AEs: 0/20; 0/20	LW: 810 nm, PD: 1.1 w/cm^2^, OP: 100 mw	*Zusanli, Dubi, Xiyan, Yinlingquan, Yanglingquan, Heding, Weizhong, Sanyinjia, Liangqiu, Xuehai, Xiguan, Taixi, Xiangu, Fengshi, Zulingqi*	15	SG: L, AC: L, BPt: L, IOD: L, SOR: L, OB: L	LA: a significantly reduction pain and stiffness and an increment in physical function.
Ling Zhao, 2010^[15]^	18/13; 60.10 ± 6.83, 59.40 ± 6.15; 2 20, 3 20	LA vs L (Nonacupoint Sham Control)	WPS: 55.01 ± 36.73; 55.19 ± 38.56 WSS: 36.17 ± 15.44; 19.95 ± 56.80 WPF: 45.92 ± 24.29; 48.41 ± 37.33 AEs: 0/20; 0/20	LW: 650 nm; 10600 nm, PD: -, OP: 36 mw; 200 mw	*Dubi*	1	SG: L, AC: U, BPt: L, IOD: L, SOR: L, OB: L	The patients of LA group have significantly greater WOMAC pain score than that in control group (49.21% and 11.99%, respectively). No side effects reported.
Mingjian Xi, 2008^[16]^	19/17; 60.10 ± 6.83, 59.40 ± 6.15; 2 20, 3 20	LA vs L	WTS: 4.05 ± 2.92; 2.29 ± 4.34 WPF 12.26 ± 7.18; 13.76 ± 7.41	LW: 650 nm; 10600 nm, PD: -, OP: 36 mw; 200 mw	*Dubi*	1	SG: L, AC: L, BPt: L, IOD: H, SOR: L, OB: L	The WOMAC score was decreased by 24.88% post treatment in the LA group, while there is an reduction by 14.32% in the control group.
Haihua Zhao, 2018^[17]^	30/30; 46–71 (mean 64), 49–72 (mean 65); 8 30, 10 30	LA + A vs A	WTS: 29/30; 24/30 AEs: 0/30; 3/30	–	*Liangqiu, Xuehai, Heding, Xiyan, Yinlingquan, Yanglingquan, Zusanli, Sanyinjiao, Juxu*	9	SG: L, AC: U, BPt: U, IOD: L, SOR: L, OB: L	LA has cured 12 patients in the LA group, while only 6 patients cured in the control group.
Li Shi, 2012^[18]^	45/44; 52.81 ± 4.05, 53.13 ± 4.14; 30 45, 28 44	LA vs A	Total effect: 43/45; 39/44 CT: 12.21 ± 3.05; 16.33 ± 4.57	LW: 840 nm, PD: –, OP: 300–500 mw	*Xuehai, Xiyan, Zusanli, yanglinguquan, Yinlingquan, dubi, Ashi point, sanyinjiao*	8	SG: L, AC: U, BPt: U, IOD: L, SOR: L, OB: L	The curing time of LA group was significantly shorter than that of control group (16.33 ± 4.57d; 12.21 ± 3.05d).
Xiangling Li, 2012^[19]^	30/30; 60 ± 10, 62 ± 8; 4 30, 4 30	A vs LA	WPS: 13/30; 5/30 WSS: 13/30; 5/30 WP: 14/30; 8/30 SSP: 11/30; 9/30 Distance: 5/30; 4/30 WPF: 8/30; 4/30 UDS: 9/30; 11/30 Squat: 7/30; 9/30 Uneven road: 10/30; 9/30 VAS score: 4; 4 (mean value)	-	*Dubi*	1	SG: U, AC: U, BPt: U, IOD: L, SOR: L, OB: L	Notably increment in the LA group in the VAS score, pain, stiffness than that of control group.
Hong Qi, 2013^[20]^	41/41; 60.87 ± 5.41, 59.46 ± 5.09; 13 41, 10 41	LA vs A + electeo-magnetic wave	PSA: 36/40; 35/38 WPS: 75.22 ± 70.62; 101.51 ± 93.02 WSS: 34.15 ± 37.25; 46.37 ± 45.65 WPF: 287.63 ± 259.43; 392.29 ± 350.60	LW: 650–660 nm; 10,600 nm, PD: 25 mw/mm^2^, OP: 36 mw; 200 mw	*Dubi*	1	SG: L, AC: L, BPt: U, IOD: L, SOR: L, OB: L	Significantly improvement of LA group in the pain, stiffness and physical function on both 2-week and 6-week post treatment. no difference in control group.
Xiumei Ren, 2010^[21]^	22/19; 61.8 ± 7.4, 59.9 ± 6.3; 4 22, 5 19	Multi L vs single L	WTS: 32.018 ± 35.151; 42.471 ± 27.662 WPS: 16.99 ± 20.26; 21.87 ± 15.25 WSS: 17.95 ± 21.68; 27.78 ± 21.83 WPF: 18.29 ± 18.91; 27.78 ± 21.83 PSA: 17/22; 12/18	LW: 650–660nm; 10,600 nm, PD: -, OP: 36 mw; 200 mw	*Dubi, Neixiyan*	2	SG: L, AC: L, BPt: L, IOD: L, SOR: L, OB: L	Significantly improvement in WOMAC score, pain, stiffness, physical function in patients of both LA group and control group on 2-week and 6-week post treatment.
Jinghua Ge, 2012^[22]^	30/30; 56.30 ± 8.03, 59.57 ± 6.52; 6 30, 6 30	LA1 vs LA2	Total effect: 24/30; 15/30 PS: 1.93 ± 0.46; 2.53 ± 0.90 SS: 1.80 ± 1.52, 1.67 ± 1.18 WP: 1.80 ± 0.96; 2.33 ± 0.76 SSP: 1.40 ± 1.07; 2.27 ± 0.87 Distance: 1.87 ± 1.04; 2.20 ± 0.61 WPF: 1.67 ± 1.30; 2.13 ± 0.51 UDS: 1.93 ± 0.64; 2.33 ± 0.76 Squat: 2.20 ± 0.96; 2.53 ± 1.04 Uneven road: 1.73 ± 1.14; 2.13 ± 0.51	LW: 650 nm; 10,600 nm, PD: -, OP: -	*Dubi*	1	SG: L, AC: U, BPt: U, IOD: L, SOR: L, OB: L	The effective rate of treatment group: 80.0% The effective rate of control group: 50.0% notably improvement in both groups before and after the treatment. significant different in the pain, walking pain, sedentary pain, physical function, up and down stair score among groups
Zhouyan Mu, 2019^[23]^	55/54; 55 ± 7, 53 ± 7; 32 55, 30 54	LA vs muscle training	Total effect: 50/55; 41/54 AEs: 2/30; 4/30 VAS score: 2.06 ± 0.36; 2.95 ± 0.47 LKSS: 86.58 ± 9.46, 76.72 ± 8.68 PT E: 80.07 ± 12.431, 68.66 ± 10.82 PT/BW E: 141.32 ± 18.48, 113.87 ± 9.85 PT F: 63.72 ± 8.47, 51.83 ± 7.36 PT/BW F: 95.83 ± 15.07, 81.42 ± 13.37 IL-6: 1.94 ± 0.34, 2.74 ± 0.51 IL-1: 45.38 ± 3.55, 53.72 ± 4.621 Sox9: 3.58 ± 0.44, 3.01 ± 0.41 Collagen II: 2.56 ± 0.47, 2.03 ± 0.43	LW: 650 nm, PD: –, OP: 0–3000 mw	*Yanglingquan*	1	SG: L, AC: U, BPt: U, IOD: L, SOR: L, OB: L	Observation group was better than the control group: IL-6 and IL-1 decreased, Sox9 and Collagen II increased. The effective rate in LA is 90.0% The effective rate in the control is 75.9% significant reduction in VAS score in both groups with the treatment group has lower value than the control group. Significant increment in LKSS score, PT, PT/BW in both groups with the treatment group own larger value than the control group, with the value of treatment group after therapy is higher. There is no serious adverse event.
Huizhu Liu, 2009^[24]^	16/16; 58.13 ± 7.81, 56.50 ± 5.80; 5 16, 4 16	Multi L vs single L	WPS: 15.96 ± 11.26; 17.56 ± 12.49 WSS: 19.38 ± 13.85; 20.91 ± 15.77 WPF: 17.55 ± 12.77; 16.46 ± 11.92 PSA: 14/16; 9/16	LW: 650 nm; 10,600 nm, PD: –, OP: –	*Dubi*	1	SG: L, AC: U, BPt: L, IOD: L, SOR: L, OB: L	There was a significant decrement in WOMAC pain, stiffness, physical function in both A and B group at all 3 time slots (2 weeks during the treatment, 6 weeks during the treatment and 4 weeks posttreatment)
Zhongchang Li, 2016^[25]^	30/30/30; 58.36 ± 11.89, 57.76 ± 11.36, 58.12 ± 11.13; 9 30, 11 30 12 30	LA vs LA + HM; LA vs HM	*LA vs. LA + HM vs. HM* VAS score: 2.74 ± 1.12; 1.87 ± 0.75, 4.07 ± 1.34 TNF-α: 56.89 ± 5.12, 44.03 ± 5.75, 45.07 ± 5.64 LKSS: 46.26 ± 5.06, 70.56 ± 9.75, 60.25 ± 9.34	–	*Neixiyan, Waixiyan, yanglingquan, Tongdian, ashi point*	5	SG: L, AC: U, BPt: U, IOD: L, SOR: L, OB: L	The VAS score of the group of applied both LA and HM is significantly lower than the other 2 groups (applied on LA or applied only HM). The knee osteoarthritis Lysholm-II score in the group of applied both LA and HM is significantly higher than the other 2 groups.
Lorna K. P. Suen, 2016^[26]^	13, 11; 71.62 ± 7.75, 73.00 ± 6.31; 0 13, 2 11	LA vs Sham LA; LA + MA vs Sham LA + MA	*Placebo LA and MA* vs *LA and placebo MA* vs *LA plus MA* vs *Placebo* NRS improvement: 4.15 ± 2.12; 4.60 ± 1.90; 5.27 ± 1.79; 5.14 ± 2.55 TUGT: 15.39 ± 4.66, 15.14 ± 5.20; 14.99 ± 4.66; 16.04 ± 7.96 KF: 125.23 ± 13.24; 123.30 ± 8.42; 130.82 ± 10.2; 126.86 ± 14.92 KE: ‐2.77 ± 3.22; −3.10 ± 2.51; −3.00 ± 2.93; −2.86 ± 3.44	LW: 650 nm, PD: 0.54 J/cm^2^, OP: 2.5 mw	*Shenmen, Subcortex, Kidney, Knee, Liver, Spleen (all in ear*)	6	SG: L, AC: U, BPt: U, IOD: L, SOR: L, OB: L	No differences in NRS, TUGT, and active/passive knee flexion and extension at baseline, post-therapy between the 4 groups. The relative differences of NRS and TUGT in subjects who received combined MAT plus LAT were higher than those treated with MAT or LAT alone, 4/6 parameters own significant within group differences in MAT and/or LAT, while no differences in placebo group
Rana S. Hinman, 2014^[35]^	65/58/64; 63.4 ± 8.7, 63.8 ± 7.5, 64.3 ± 8.6; 43 71, 31 70, 38 70	LA vs Sham LA; LA vs A	*LA vs. Sham LA:* WPFS: 21.9 ± 12.3; 21.7 ± 12.0 WPS: 6.6 ± 3.9; 6.6 ± 3.9 WP: 3.6 ± 2.4; 3.7 ± 2.6 SP: 3.3 ± 2.4; 2.9 ± 2.4 VAS score: 3.4 ± 2.2; 3.4 ± 2.3 AQoL-6D: 0.73 ± 0.17; 0.78 ± 0.12 SF-12 PCS: 39.4 ± 9.5; 40.2 ± 10.1 SF-12 MCS: 53.0 ± 9.9; 53.2 ± 10.4 *LA vs. A:* WPFS: 21.9 ± 12.3; 22.5 ± 13.1 WPS: 6.6 ± 3.9; 6.7 ± 3.8 WP: 3.6 ± 2.4; 3.4 ± 2.2 SP: 3.3 ± 2.4; 3.2 ± 2.3 VAS score: 3.4 ± 2.2; 3.3 ± 2.2 AQoL-6D: 0.73 ± 0.17; 0.75 ± 0.18 SF-12 PCS: 39.4 ± 9.5; 40.7 ± 9.6 SF-12 MCS: 53.0 ± 9.9; 51.5 ± 11.0	–	*–*	–	SG: L, AC: L, BPt: L, IOD: H, SOR: L, OB: L	Neither needle nor laser acupuncture significantly improved pain or function compared with sham at 12 weeks. Compared with control, needle and laser acupuncture resulted in modest improvements in pain at 12 weeks, but not at 1 year. Needle acupuncture resulted in modest improvement in function compared with control at 12 weeks but not significantly different and not maintained at 1 year. No differences for most secondary outcomes and no serious adverse events.
Ali Lafta Mezaal, 2018^[36]^	23/23; 56.65 ± 8.568, 57.30 ± 6.852; 5 23, 6 23	LA vs L (Nonacupoint Sham Control)	WOMAC improvement: 29.78 ± 13.79; 32.39 ± 12.8 McGill improvement: 20.13 ± 8.79 19.56 ± 5.77 MSS: 12.2174 ± 4.899; 12.000 ± 3.438 NPRS improvement: 3.95 ± 1.364; 3.82 ± 1.26 KF improvement: 3.737 ± 2.82; 5.55 ± 3.39 KE improvement: 1.475 ± 0.783; 1.38 ± 0.783 RM: 115; 119.0000 ± 6.22312	LW: 830 nm, PD: 30 mw/cm^2^, OP: 3 mw	*Xuehai, Liangqiu, Yinlingquan, Dubi, Yanglingquan, Xiyan*	6	SG: L, AC: U, BPt: L, IOD: L, SOR: L, OB: L	A significant pain reduction by NRS and McGill; an increase in functional activity by WOMAC, range of motion in 3 study groups after 10 sessions. Significant difference in pain reduction among 3 groups The post hoc Least Significant Difference (LSD): the largest improvement was in Group I. Functional activity: the largest improvement was in Group II. No significant difference among groups: active knee flexion and extension.
Xueyong Shen, 2008^[27]^	24/24; range of 50–70, range of 50–70; -	Multi L vs single L	WOMAC scores PGA	LW: 650 nm; 10,600 nm, PD: –, OP: –	*Dubi*	1	SG: L, AC: U, BPt: U, IOD: L, SOR: L, OB: L	No difference in WOMAC scores, patients’ global assessment before and after treatment. Patients in both groups were reduced significantly compared to baseline by the end of the first course in the WOMAC scores of patients.
Fang-Yin Liao, 2020^[28]^	15/15; 70.53 ± 6.89, 69.73 ± 6.91; 1 15, 1 15	LA vs Sham LA	LI VAS score PPT	LW: 780 nm; 830 nm, PD: –, OP: 50 mw; 30 mw	*Yinlingquan, Xuehai, Heding*	3	SG: L, AC: U, BPt: L, IOD: L, SOR: L, OB: L	A notably reduction of LA group in the Lequesne index, conscious VAS 4 weeks after treatment and an increment in PPT in LA group than the placebo group
Lin Lin, 2020^[29]^	84/55; 61.25 ± 5.55, 64.73 ± 6.92; 18 84, 16 55	LM vs M	WPS: 143.33 ± 84.89; 239.93 ± 123.51 WSS: 44.40 ± 33.60; 18 (11, 32) WPFS: 299 (176.75, 531.50); 216 (117, 395) PF: 67.5 (55, 80); 65 (53.75, 75) RP: 25 (0, 50); 25 (0, 75) RE: 33.33 (0, 100); 66.67 (0, 100) VT: 50 (40, 60); 60 (45, 70) MH: 64 (56, 75); 68 (60, 76) SF: 75 (62.5, 84.38) 75 (62.5, 87.5) BP: 67.5 (55, 67.5) 67.5 (57.5, 77.5) GH: 40 (31.25, 55) 55 (35, 65)	LW: 10,600 nm, PD: 61.2–68.8 J/cm^2^, OP: 160–180 mw	*Dubi, Neixiyan, Ashi point*	3	SG: L, AC: U, BPt: L, IOD: L, SOR: L, OB: L	Only difference: significantly more benefit in laser moxibustion group than traditional moxibustion in physical function at the follow-up of 4 weeks post treatment.
Zhao Lin, 2020^[30]^	201/191; 63.5 ± 7.67, 63.1 ± 6.0; 48 201, 50 191	LA vs Sham LA	WPS: 143.33 ± 84.89 COMP: 270.2 (240.7305.9); 301.0 (260.2, 364.3) IL-1β: 2.5 (0.8,5.7); 1.3 (0.6, 3.67) IL-2: 33.2 (15.0, 76.8); 16.8 (8.4,68.3) IL-6: 4.6 (2.5,9.8); 4.7 (2.1,13.6) IL-8: 50.8 (17.0,170.4); 25.2 (10.0, 62.3) MCP-1: 110.4 (89.1140.5); 111.4 (82.7150.7) MMP-3: 6.2 (4.1,12.3); 7.0 (4.6,9.8) MMP-13: 206.7 (132.8275.8); 120.8 (42.4245.4) WPSC: 2.9 (1.1, 5.7); −0.2 (-1.4, 1.6) WSSC: 3.4 (0, 6.5); 0 (-1.8, 1.4) WPFSC: 14.7 (4.0, 25.2); −0.6 (-5.7, 6.6) VASC: 34.0 (22.0, 45.0); 3.0 (-3.5, 20.0) RF: 75.0 (60.0,90.0); 65.0 (55,75) RP: 75.0 (0,100.0); 0 (0,75.0) BP: 68.0 (58.0,78.0); 58.0 (45.0,68.0) GH: 50.0 (40.0,65.0); 50.0 (40.0,60.0) VT: 60.0 (50.0,70.0); 55.0 (45.0,70.0) SF: 75.0 (63.0,88.0); 63.0 (50.0,88.0) RE: 100.0 (83.5100.0); 100.0 (0, 100.0) MH: 68.0 (60.0,80.0); 68.0 (60.0, 80.0) SF36 PCS: 44.8 (36.0,50.0); 38.1 (31.6, 44.3) SF36MCS: 50.7 (45.9,55.7); 50.4 (39.7,55.5) AEs:24/201, 6/191	LW: 10,600 nm, PD: 61.2–68.8 J/cm^2^, OP: 160–180 mw	*Dubi, Ashi point*	2	SG: L, AC: L, BPt: L, IOD: L, SOR: L, OB: L	In the LA group, the median WOMAC pain score fell considerably by week 4. In comparison to the sham laser, LA treatment resulted in considerable pain relief and improved function at week 24. From week 4 through week 24, the physical component of the quality of life in the LA group outperformed the sham control group.
Chiung-Hui Huang, 2020^[31]^	41/541 73.10 ± 7.37, 73.20 ± 8.28; 13 39, 15 40	LA vs Sham LA	WPS: 143.33 ± 84.89 WSS: 1.03 ± 0.90, 1.53 ± 0.85 KF: 99.1 ± 7.51, 97.25 ± 6.4 AEs:0/41, 0/41	LW: 808 ± 10 m nm, PD: –, OP: 300 mw	*Neiguan, Sanyinjiao, Taixi, Kunlun, Fengshi, and Futu*	6	SG: L, AC: L, BPt: L, IOD: L, SOR: L, OB: L	On days 2 and 3, the LA group had better joint flexion and reduced stiffness than the control group.

*Denotation*: outcome measurements consist of either continuous data (M ± SD[T]; M ± SD[C]) or dichotomous data (improvement patients no./overall patients[T]; improvement patients no./overall). Patients(C).

50 foot w = 50 foot walking distance, AEs = adverse events, AR = activity restriction, SF-36 form parameters, BP = body pain, C = control group, CAS = Credibility Expectancy Affectively-Based Expectancy Scale (Q4–6), CCS = Credibility/Expectancy Cognitively Based Credibility Scale (Q1–3), CT = curing time, CTFS = Credibility/Expectancy Think & Feel Scale (Q1–6), GH = General Health, HM = Herbal Medicine, IL-1 = serum interleukin-1, IL-6 = serum interleukin-6, KC = knee circumference, KE = knee extension, KF = knee flexion, LA = Laser Acupuncture, LI = Lequesne index, LKSS = Lysholm Knee Scoring Scale (LKSS), M = male, MAS = McGill Affective Scale (Q12–15), MH = Mental Health, MHLCDS = Multi-dimensional Health Locus of Control Form C (MHLC-C)–Doctor Scale (Q3, 5, 14), MHLCOPS = MHLC-Other People Scale (Q7, 10, 18), MHLCPO = Multi-dimensional Health Locus of Control Form C (MHLC-C)-Powerful Others (Q3, 5, 7, 10, 14, 18), MHILCC = Multi-dimensional Health Locus of Control Form C (MHLC-C)–Chances (Q2, 4, 9, 11, 15, 16), MHLCIB = Multi-dimensional Health Locus of Control Short Form C (MHLC-C)–Internal Belief (Q1, 6, 8, 12, 13, 17), MSA = McGill Sensory & Affective (Q1–15), MSS = McGill Sensory Scale, MTS = medial tenderness score of the knee, NHP = Nothingham Health Profile total score, NPRS = numeric pain rating scale, NRS = numerical rating scale, PGA = patients’ global assessment, PPI = McGill Pain Questionnaire Present Pain Intensity (Q17), PPT = pain pressure threshold, PF = Physical Functioning, PS = pain score, PSA = Patients’ self-assessment, PT E = peak torque of the extensors, PT F = peak torque of the flexors, PT/BW E = peak torque/body weight of the extensors, PT/BW F = peak torque/body weight of the flexors, RE = role-emotional, RM = range of motion, RP = Role-Physical, SF = Social Functioning, SP = standing pain, SKFS = Saudi knee function scale, SS = stiffness score, SSP = sedentary standing pain, T = treatment group, TNF-α = tumor necrosis factor-alpha, TUGT = timed-up and-go test, UDS = up and down stairs, VAS = visual analogue scale, VT = Vitality, WAIA = WAI (C) All Scales (Task, Goal & Bond), WAIBS = Working Alliance Inventory Short Form (WAI-C)–Bond Scale Q3, 5, 7 & 9, WAIG = Working Alliance Inventory Short Form (WAI-C)–Goals (Q4, 6, 10 & 11), WAIT = Working Alliance Inventory (WAI) Short Form (C)–Task (Q1, 2, 8, 12), WOMAC = Western Ontario and McMaster Universities, WP = walking pain, WPHS = WOMAC physical function score, WPS = WOMAC pains core, WSS = WOMAC stiffness score, WTS = WOMAC total score.

Laser parameters categories: LW = laser wavelength, OP = output power, PD = power density.

Risk of bias categories: AC = allocation concealment, BPt = blinding of participants/personnel, IOD = incomplete outcome data, OB = others bias(baseline), SG = sequence generation, SOR = selective outcome reporting. Risk of bias judgements: L = low risk, H = high risk, U = unclear risk.

#### 2.2.3. Study participants

The 25 RCTs comprised a total of 2075 participants. Every participant received a KOA diagnosis. Participants’ ages ranged from 18 to 85 years old, and KOA durations varied from 4 weeks to 30 years.

#### 2.2.4. Study intervention

Comparisons of interest is the LA versus. Sham LA (efficacy), LA versus. A (comparative effectiveness), LA combined with A versus A (effectiveness as an adjunct), and any other paper used LA in their treatment. Laser acupuncture was applied in all 25 RCTs.

#### 2.2.5. Study outcome measures

Apart from key outcome measurements of WOMAC total score (WTS), WOMAC pains core (WPS), WOMAC stiffness score (WSS), WOMAC physical function score, patient self-assessment (PSA), visual analogue scale (VAS), other parameters including Lequesne index (LI), patients’ global assessment, knee extension, knee flexion (KF), range of motion, curing time, walking pain, sedentary standing pain, up and down stairs, Saudi knee function scale, numeric pain rating scale, adverse events (AEs), pain pressure threshold, Nothingham Health Profile total score, 50 foot walking distance, knee circumference, medial tenderness score of the knee, McGill Sensory Scale (MSS), McGill Affective Scale, McGill Sensory & Affective, McGill Pain Questionnaire Present Pain Intensity, Credibility/Expectancy Cognitively Based Credibility Scale, Credibility Expectancy Affectively-Based Expectancy Scale, Credibility/Expectancy Think & Feel Scale, WAI-Task (WAIT), WAI-Bond Scale, WAI-Goals, WAI All Scales (WAIA), MHLC-Internal Belief, MHLC-Chances(MHLCC), MHLC-Powerful Others, MHLC–Doctor Scale, MHLC-Other People Scale, PSA, pain score (PS), stiffness score, peak torque of the extensors, peak torque of the flexors (PT F), peak torque/body weight of the extensors, peak torque/body weight of the flexors, Lysholm Knee Scoring Scale (LKSS), serum interleukin-6, serum interleukin-1, timed-up and-go test, tumor necrosis factor-alpha, numerical rating scale, walking pain, standing pain, activity restriction, and SF-36 form parameters which comprises the following: Social Functioning (SF), Role-Emotional, Mental Health, Beta-endorphin, Substance P, General Health, body pain, and Physical Functioning. We set the WTS as the primary outcome and the others as the secondary outcomes.

### 2.3. Data extraction and management

A premade data extraction table was used independently by 2 reviewers to gather information from the trials that were included. Comparisons of interest is the LA versus. Sham LA (efficacy), LA versus. A (comparative effectiveness), LA combined with A versus A (effectiveness as an adjunct), and any other paper used LA in their treatment. The general information, study design, methods, participants, interventions, outcomes, laser parameters, and acupoints utilized were the main components of the data extraction table. The 2 reviewers discussed and worked out any differences (R.H and CX.G). If there was any disagreement in the discussion, the mediator, Professor Hu, was invited to make arbitration and resolve the issue.

### 2.4. Risk of bias assessment

**Figure 3.**
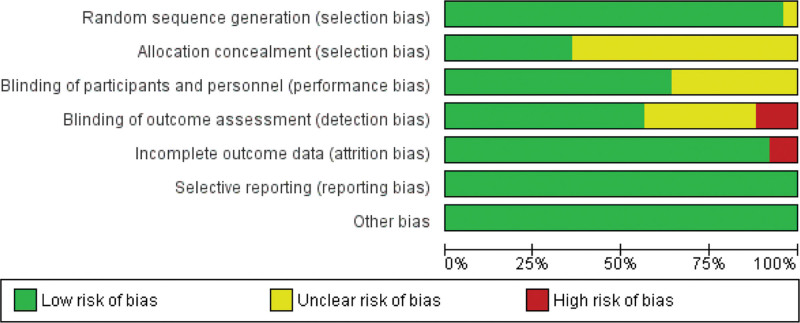
Graph of risk of bias assessment.

**Figure 4.**
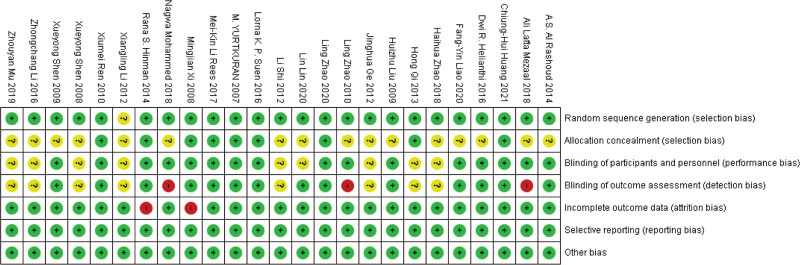
Summary of risk of bias assessment.

Two reviewers independently assessed the included studies using the PRISMA guideline to determine the risk of bias.^[12]^ The 7 criteria used to assess bias in twenty-five studies: generating a random sequence, hiding the allocation, blinding the staff and participants, and blinding the outcome evaluation, partial outcome data, selective reporting, and other potential sources of bias. Each category’s risk of bias was rated as low (L), unclear, or high (H). The 2 reviewers had a discussion prior to any disagreements. If any disagreement was made in the discussion, the mediator (Prof Hu) was invited to make arbitration and resolve the issue. We had planned to analyze potential publication bias using funnel plots if the meta-analysis contained ten or more studies. The graph and summary of the risk of bias assessment are displayed in Figure 3 and Figure 4 separately.

### 2.5. Statistical analysis

Revman V.5.0 software was used for meta-analysis. Methods were based on Cochrane Handbook5.1.0. The analysis method’s inverse variance was used with an interval of confidence of 95%. Risk difference and risk ratio analyses were performed on dichotomous data. Continuous data were analyzed using mean difference (MD) with standard deviation. The random effect model was applied. When *P* < .05, it indicates there is a significant difference between groups. The I^2^ is the proportion of heterogeneity which indicated: (1) 0% to 40% may not be necessary; (2) There could be moderate heterogeneity between 30% and 60%; (3) there could be substantial heterogeneity between 50% and 90%; (4) There could be significant heterogeneity in 75% to 100%.^[39]^ Intention-to-treat analysis was used to examine missing data from the original trials. All analyses were based on the available published data. Data was pooled with the same measure scale. If ten or more studies were included in the meta-analysis, a funnel plot was used to analyze potential publication bias.

## 3. Results

**Figure 5.**
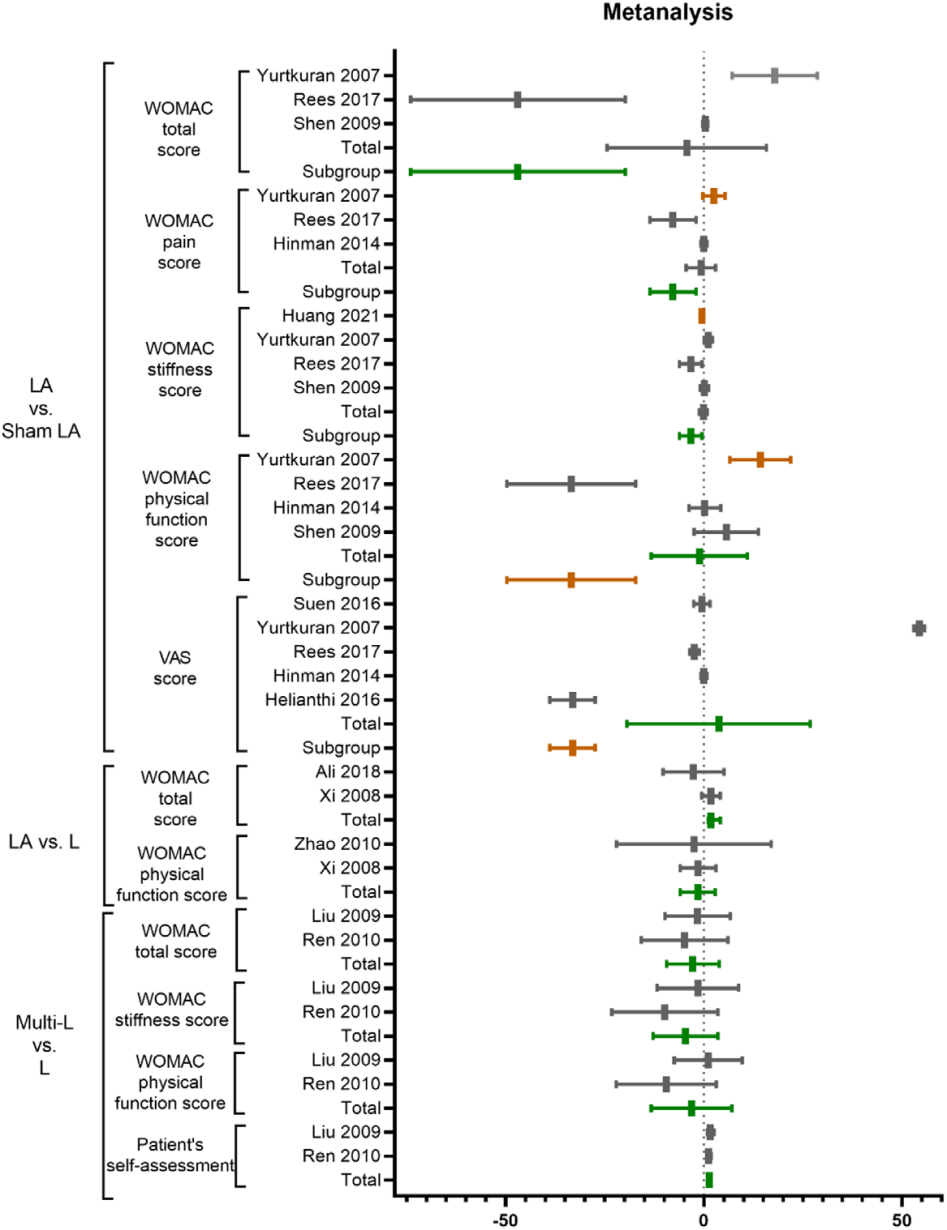
The forest plot of laser acupuncture with different comparison groups.

**Figure 6.**
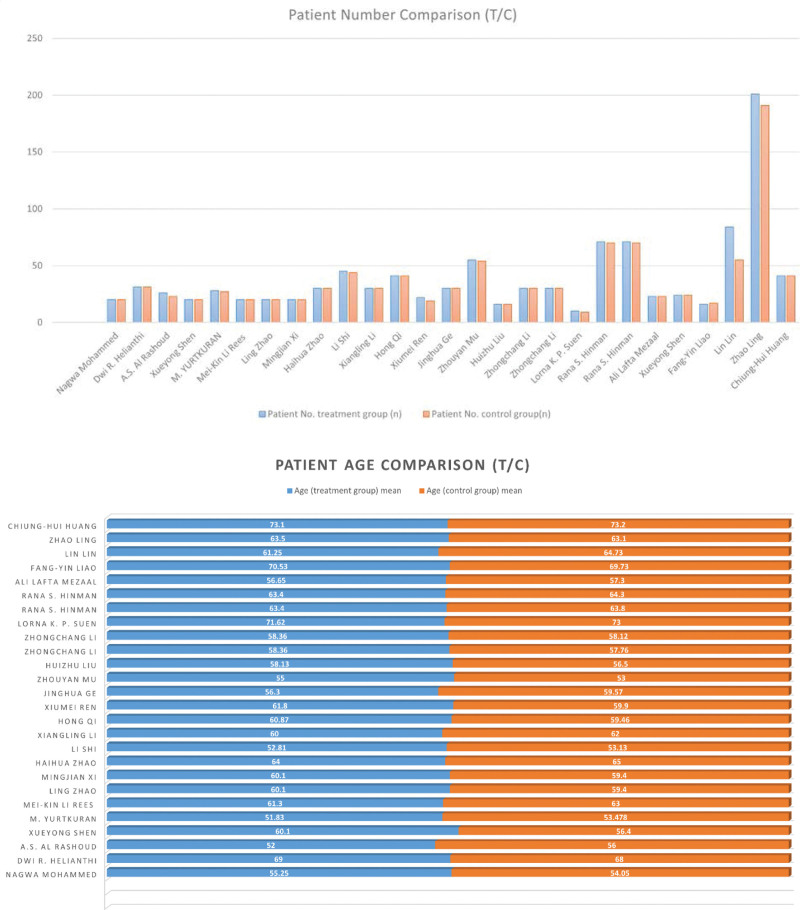
An outline of patient number, age in included studies.

Meta-analyses were conducted in the following outcomes: LA combined with A versus LA. In addition, other studies were chosen, LA versus Sham LA, LA versus A, LA versus L, and multi-L versus L, as study designs were also analyzed if data were available. The results are shown in Figure 5 which listed a whole forest plot of laser acupuncture with different comparison groups. Table 2 demonstrated a classification category of outcome measurements. Table 3 outlined average, minimum, and maximum of included patient number, age, and laser parameters. Figure 6 outlined age and patient number distribution of included studies (Fig. 6).

**Table 2:** Classification category of outcome measurements.

Category	Parameters
WOMAC	WTS: WOMAC total score WPS: WOMAC pains core WSS: WOMAC stiffness score WPHS: WOMAC physical function score
McGill	MSS: McGill Sensory Scale MAS: McGill Affective Scale (Q12–15) MSA: McGill Sensory & Affective (Q1–15) PPI: McGill Pain Questionnaire Present Pain Intensity (Q17) CCS: Credibility/Expectancy Cognitively Based Credibility Scale (Q1–3) CAS: Credibility Expectancy Affectively-Based Expectancy Scale (Q4–6) CTFS: Credibility/Expectancy Think & Feel Scale (Q1–6)
Biochemical cytokines	IL-6: serum interleukin–6 IL-1: serum interleukin–1 TUGT: Timed-up and-go test TNF-α: Tumor necrosis factor-alpha Substance P Beta-endorphin
SF-36	PF: Physical Functioning RP: Role-Physical BP: Body Pain GH: General Health VT: Vitality SF: Social Functioning RE: Role-Emotional MH: Mental Health
WAI	WAIT: Working Alliance Inventory (WAI) Short Form (C)–Task (Q1, 2, 8, 12) WAIBS: Working Alliance Inventory Short Form (WAI-C)–Bond Scale Q3, 5, 7 & 9 WAIG: Working Alliance Inventory Short Form (WAI-C)–Goals (Q4, 6, 10 & 11) WAIA: WAI (C) All Scales (Task, Goal & Bond)
MHLC	MHLCIB: Multi-dimensional Health Locus of Control Short Form C (MHLC-C)–Internal Belief (Q1, 6, 8, 12, 13, 17) MHILCC: Multi-dimensional Health Locus of Control Form C (MHLC-C)–Chances (Q2, 4, 9, 11, 15, 16) MHLCPO: Multi-dimensional Health Locus of Control Form C (MHLC-C)–Powerful Others (Q3, 5, 7, 10, 14, 18) MHLCDS: Multi-dimensional Health Locus of Control Form C (MHLC-C)–Doctor Scale (Q3, 5, 14) MHLCOPS: MHLC-Other People Scale (Q7, 10, 18)
Patients	PSA: Patients’ self-assessment PGA: patients’ global assessment AEs: adverse events
Others rating scales	LKSS: Lysholm Knee Scoring Scale (LKSS) LI: Lequesne index, VAS: visual analogue scale NHP: Nothingham Health Profile total score NRS: Numerical rating scale SKFS: Saudi knee function scale NPRS: numeric pain rating scale
Muscle assessment	PT E: peak torque of the extensors PT F: peak torque of the flexors PT/BW E: peak torque/body weight of the extensors PT/BW F: peak torque/body weight of the flexors KE: knee extension KF: knee flexion
Others	PS: pain score SS: stiffness score WP: walking pain SP: standing pain AR: Activity restriction RM: range of motion CT: curing time SSP: sedentary standing pain UDS: up and down stairs PPT: pain pressure threshold 50 foot w: 50 foot walking distance KC: knee circumference MTS: medial tenderness score of the knee

**Table 3:** Statistics of included patients and laser parameters.

	Total patient no.	Age (T/C)	Laser power
Minimum value	19^[26]^	52.81 ± 4.05/53.13^[18]^	2.5 mw^[26]^
Middle value	33/31	59.92/60.60	36 mw + 200 mw^[15,16,23,24]^
Maximum value	139^[29]^	71.62 ± 7.75/73^[26]^	3000 mw^[23]^

### 3.1. LA versus Sham LA (efficacy)

**Figure 7.**
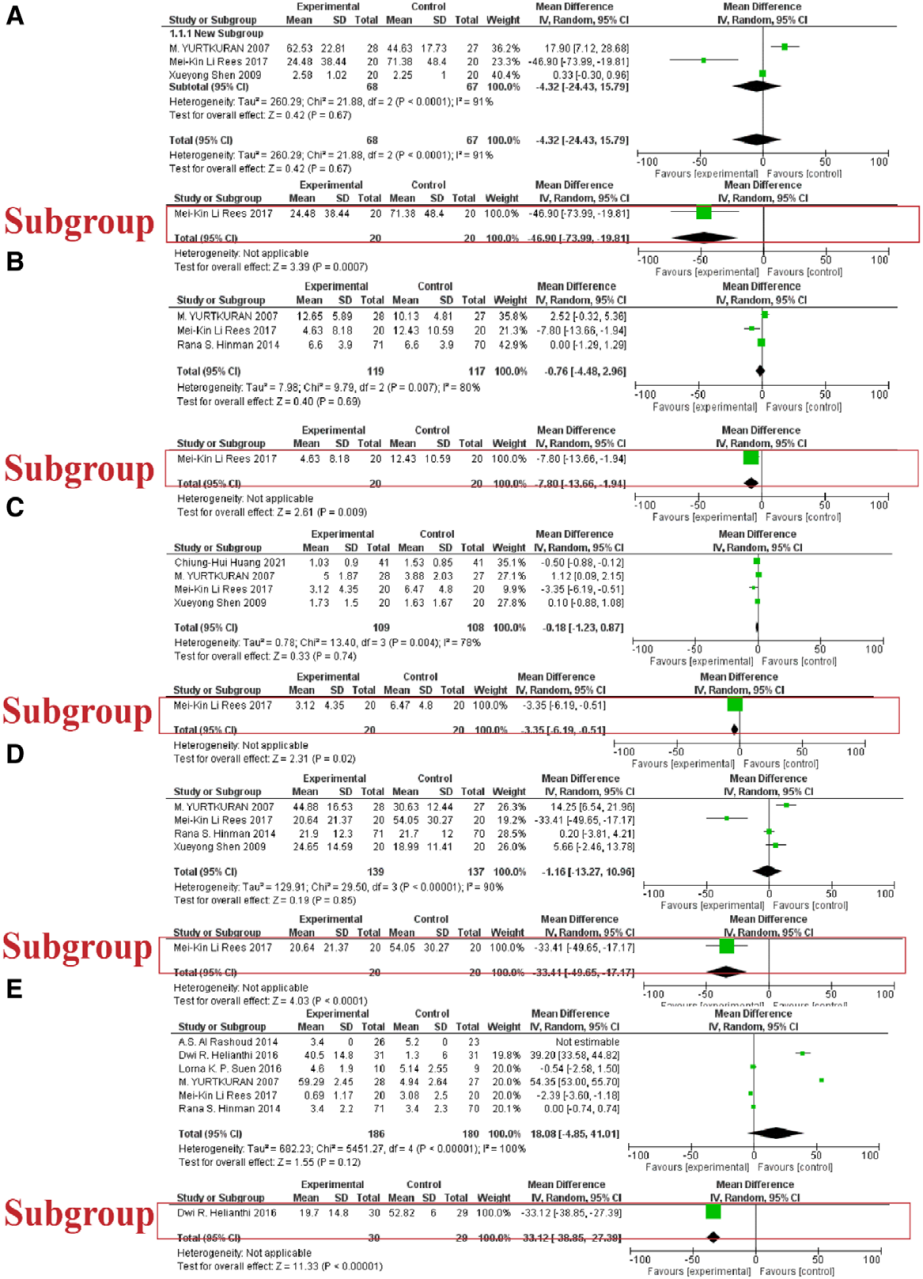
The statistical analysis of LA versus sham LA. (A) WOMAC total score of the group LA versus sham LA and subgroup with effective wavelength (810 nm); (B) WOMAC pain score of the group LA versus sham LA and subgroup with effective wavelength (810 nm); (C) WOMAC stiffness score of the group LA versus sham LA and subgroup with effective wavelength (810 nm); (D) WOMAC physical function score of the group LA versus sham LA and subgroup with effective wavelength (810 nm); (E) VAS score of the group LA versus sham LA and subgroup with effective wavelength (785 nm).

Ten included studies (562 patients) compared LA with the sham LA.^[14,26,28,31–35,37,38]^ Figure 7 listed statistical data of LA versus sham LA.

#### 3.1.1. WOMAC total score

Three studies reported the WTS.^[14,37,38]^ The statistical analysis produced a nonsignificant MD (MD: ‐4.32 [‐24.43, 15.79], I^2^ = 91%). Subgroup analysis with 810nm effective laser wavelength showed a significant result (MD: ‐46.90 [‐73.99, ‐19.81]), indicating that the LA is more effective than the sham control group by using 810 nm-wavelength laser parameters in assessing the WTS (Fig. 7A).

#### 3.1.2. WOMAC pain score

Three studies reported the WOMAC pain score.^[35,37,38]^ The pooling showed non-significant LA results compared to the sham LA alone (MD: ‐0.76 [‐4.48, 2.96], I^2^ = 80%). The subgroup with effective wavelength (810 nm) showed a significant outcome (MD: ‐7.80 [‐13.66, ‐1.94]) of LA compared with sham LA in evaluating the WOMAC pain score (Fig. 7B).

#### 3.1.3. WOMAC stiffness score

Four studies reported the WOMAC stiffness score outcome.^[14,31,37,38]^ The pooling showed non-significant LA results compared to the sham LA alone (MD: ‐0.18 [‐1.23, 0.87], I^2^ = 78%). The subgroup with effective wavelength (810nm) showed a significant outcome (MD: ‐3.35 [‐6.19, ‐0.51]) of LA compared with sham LA in evaluating the WOMAC stiffness score (Fig. 7C).

#### 3.1.4. WOMAC physical function score

Four studies reported the WOMAC physical function score outcome.^[14,35,37,38]^ The pooling showed non-significant LA results compared to the sham LA alone (MD: ‐1.16 [‐13.27, 10.96], I^2^ = 90%). The subgroup with effective wavelength (810nm) showed a significant outcome (MD: ‐33.41 [‐49.65, ‐17.17]) of LA compared with sham LA in evaluating the WOMAC physical function score (Fig. 7D).

#### 3.1.5. MCID pass rate

Based on the results of previous research, the minimum clinically relevant difference (MCID) for the WOMAC was found to be 4.2 for the PS, 1.9 for the stiffness score, 10.1 for the physical function score, and 16.1 for the total score.^[40]^ Patients with LA had higher MCID attainment rates for the physical function score, PS, stiffness score, and overall WOMAC score than did Sham LA patients. The WTS was 26.50% in Sham LA compared to 23.90% in LA; the WOMAC pain score was 18.70% in Sham LA and 3.65% in LA; the WOMAC stiffness score was 56.00% in Sham LA compared to 54.63% in Sham LA; and the WOMAC stiffness score was 56.00% in Sham LA compared to 54.63% in Sham LA. In LA, the WOMAC physical function score was 20.14%, while in Sham LA, it was 14.60%.

#### 3.1.6. VAS score

Six studies reported the VAS score outcome.^[26,33–35,37,38]^ The pooling showed non-significant LA results compared to the sham LA alone (MD: 18.08 [‐4.85, 41.01], I^2^ = 100%). The subgroup with effective wavelength (785 nm) showed a significant outcome (MD: ‐33.12 [‐38.85, ‐27.39]) of LA compared with sham LA in evaluating the VAS score (Fig. 7E).

#### 3.1.7. Others

In biochemical analysis, Nagwa Mohammed et al found that LA significantly improved serum beta-endorphin and substance P levels.^[32]^ In behavioral analyses, Dwi R. Helianthi et al found that the LI index significantly improved in LA than in sham LA.^[33]^ Chiung-Hui Huang et al found that KF was improved after LA than sham LA.^[31]^ Fang-Yin Liao evaluated that LI and pain pressure threshold scores were improved in LA than in sham control.^[28]^ Though slightly, there is an improvement in AQoL-6D, SF-12 PCS, and SF-12 MCS in Rana S. Hinman study.^[35]^ Lorna K. P. Suen et al found that a combination of LA and manual acupuncture (MA) is more effective than any single one.^[26]^ Mei-Kin Li Rees, in her dissertation, also demonstrated that there is an improvement in MSS, McGill Affective Scale, MSA, McGill Pain Questionnaire Present Pain Intensity, Credibility/Expectancy Cognitively Based Credibility Scale, Credibility Expectancy Affectively-Based Expectancy Scale, Credibility/Expectancy Think & Feel Scale, WAI-Task, WAI-Bond Scale, WAI-Goals, WAI All Scales, MHLC-Internal Belief, MHLCC, MHLC-Powerful Others, MHLC–Doctor Scale, and MHLC-Other People Scale in the group of LA than sham LA.^[38]^

### 3.2. LA versus A (comparative effectiveness)/LA versus A + electromagnetic wave

There were 3 studies included comparing LA with the A alone.^[18,19,35]^ The study showed that LA and A can improve the VAS score, pain, and morning stiffness improvement rate significantly compared to the sham control group.^[19]^ Acupuncture with needles and lasers produced pain relief without any major side effects as compared to the control group.^[35]^

The physical function score, stiffness score, PS, and overall score of the WOMAC are unknown in the LA versus A comparison group because there was only one paper and all of its findings were unsuccessful, and there was insufficient data in the original paper to combine and analyze. In order to meet MCID, there were no participants in either group, according to the one published paper.

One study compared the LA with the combination of acupuncture and electromagnetic waves, which shows that by using multi-laser treatment, there is a notable improvement in the physical function score, pain, and stiffness, being equivalent compared with acupuncture plus electromagnetic wave therapy.^[20]^ The study demonstrated an almost equivalent effect of LA and the combination of A and electromagnetic waves.^[20]^

### 3.3. LA + A versus A versus A + Sham LA (effectiveness as an adjunct)

There is 1 study that suggested that using LA plus A could have a faster curing time than A alone.^[17]^ Besides, the difference among their total effects was not significant.^[17]^ On top of that, the AEs adverse events in the experimental group were 0, while the control group had 3 cases of transient abdominal pain.^[17]^ Another study compared the LA plus A versus. Deactivated LA plus A, the value of the numerical rating scale and timed-up and-go test in subjects who received combined acupuncture (A) plus laser acupuncture (LA) were higher than the group p of the combination of deactivated LA and MA.^[26]^

### 3.4. LA versus L

**Figure 8.**
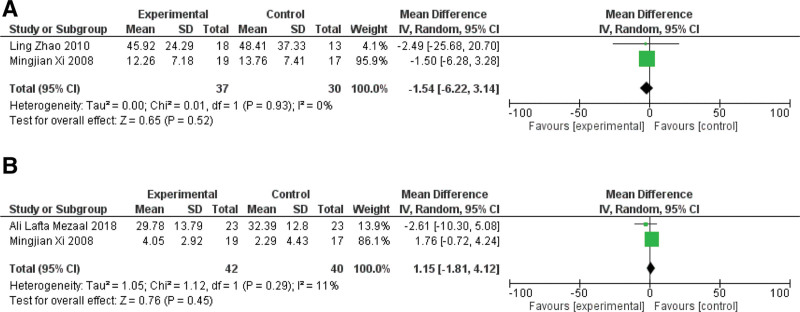
The statistical analysis of LA versus L. (A) WOMAC total score of the group LA versus L; (B) WOMAC physical function score of the group LA versus L.

Three studies (including 122 patients) made the contrast between LA and L in the nonacupoint area.^[15,16,36]^ They used only laser as the control group, which is non-acupoint sham control.^[15,16,36]^ Figure 8 listed statistical data of LA versus L.

#### 3.4.1. WOMAC total score

Two studies reported the WTS outcome in LA compared with A.^[15,16]^ The pooling results showed non-significant LA results compared to the sham LA alone (MD: ‐1.54 [‐6.22, 3.14], I^2^ = 0%), showing that the effectiveness of LA versus L on non-acupoints is almost equivalent, and they both eliminate the syndrome of patients with knee osteoarthritis. Meanwhile, they both use a combined 650 nm and 10.6 um laser wavelength (Fig. 8A).

#### 3.4.2. WOMAC physical function score

Two studies reported the WOMAC physical function score outcome in LA compared with A.^[16,36]^ The pooling results showed non-significant LA results compared to the sham LA alone (MD: 1.15 [‐1.81, 4.12], I^2^ = 11%), showing that the effectiveness of LA versus L on non-acupoints is almost equivalent, and they both eliminate the syndrome of patients with knee osteoarthritis (Fig. 8B).

#### 3.4.3. MCID pass rate

We further analyzed their MCID attainment rate for WPS, WSS, WPFS. Patients with LA had a higher MCID attainment rate for WPS, WSS, WPFS than patients with L. We further compared the WOMAC MCID rates between the LA group and the control group and found that in the LA versus L comparison, the WTS attainment rate is 36.5% in LA and 36.51% in L. The WOMAC pain score attainment rate is 28.57% in LA and 20.63% in L. The WOMAC stiffness score attainment rate in LA and 20.63% in L is higher than those in L. WSS and WPFS in LA are higher than those in L. LA’s WOMAC stiffness score is 28.57% and L is 20.63%. LA’s WOMAC physical function score is 28.57%, L is 20.63%.

#### 3.4.4. Others

In the study, Ali Lafta Mezaal et al showed that in knee extension, MSS, and numeric pain rating scale improvement, the LA exerts a better effect than the laser only.^[36]^ However, the range of motion, KF, MCGILLL improvement parameters, and LA are less effective than laser irradiation.^[36]^

### 3.5. Multi-L versus L

**Figure 9.**
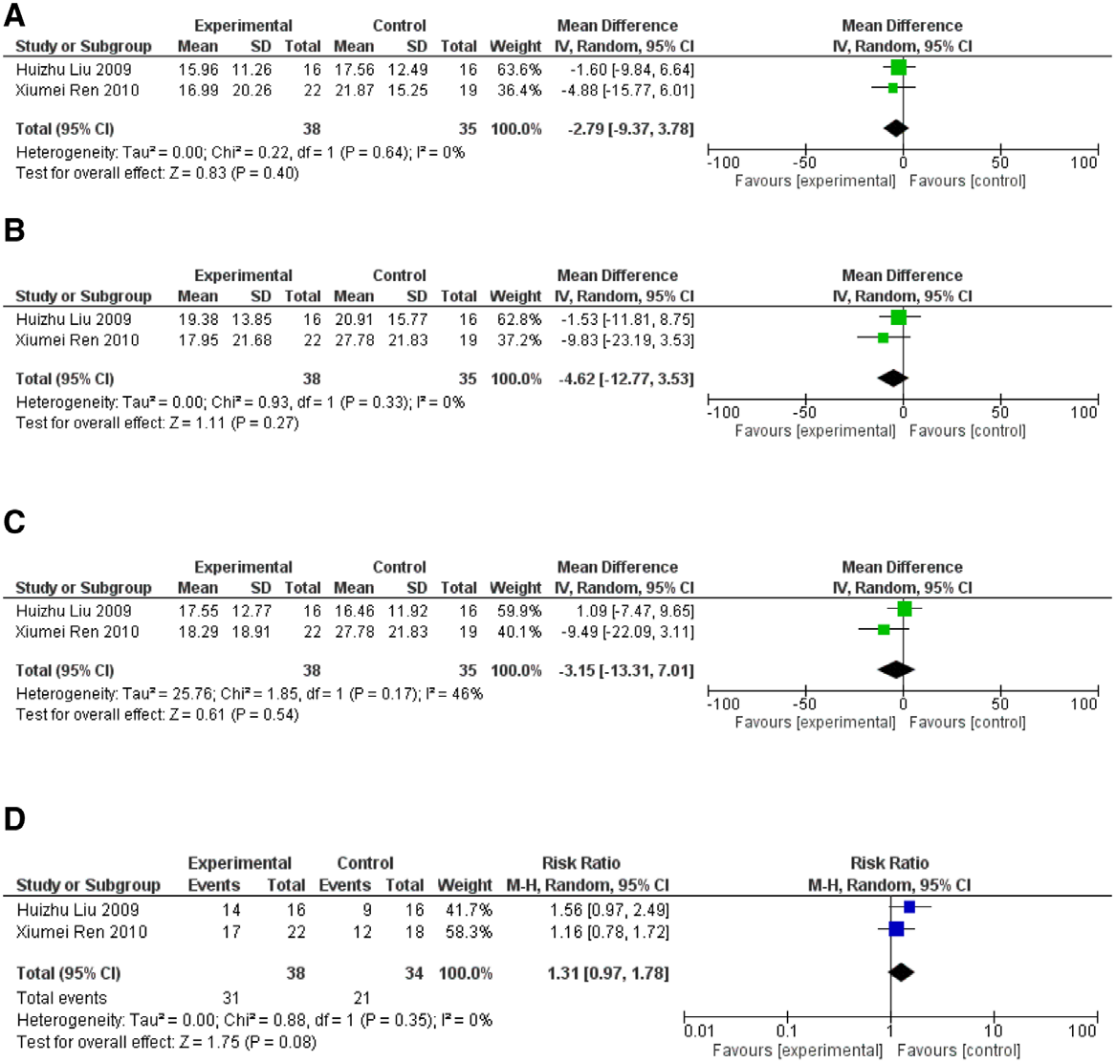
The statistical analysis of Multi-L versus single-L. (A) WOMAC pain score of the group Multi-L versus single-L; (B) WOMAC stiffness score of the group Multi-L versus single-L; (C) WOMAC physical function of the group Multi-L versus single-L (D) patient’s self-assessment score of the group Multi-L versus single-L.

There were 3 studies (including 121 patients) showed the contrast of using multi-lasers (multi-L) compared with a single source of laser (L).^[21,24,27]^ Figure 9 listed statistical data of multi-L versus single-L group.

#### 3.5.1. WOMAC pain score

Two studies reported the WOMAC pain score outcome in multi-L compared with L.^[21,24]^ The pooling results showed non-significant multi-L results compared to the L alone (MD: ‐2.79 [‐9.37, 3.78], I^2^ = 0%), showing that the effectiveness of multi-L versus L is almost equivalent, and they both eliminate the syndrome of patients with knee osteoarthritis. Their wavelengths combine 0.65 to 0.66 μm and 10.6 μm (Fig. 9A).

#### 3.5.2. WOMAC stiffness score

Two studies reported the WOMAC stiffness score outcome in multi-L compared with L.^[21,24]^ The pooling results showed non-significant multi-L results compared to the L alone (MD: ‐4.62 [‐12.77, 3.53], I^2^ = 0%), showing that the effectiveness of multi-L versus L is almost equivalent, and they both eliminate the syndrome of patients with knee osteoarthritis. Their wavelength combines 0.65 to 0.66 μm and 10.6 μm (Fig. 9B).

#### 3.5.3. WOMAC physical function score

Two studies reported the WOMAC physical function score outcome in multi-L compared with L.^[21,24]^ The pooling results showed non-significant multi-L results compared to the L alone (MD: ‐3.15 [‐13.31, 7.01], I^2^ = 46%), showing that the effectiveness of multi-L versus L is almost equivalent, and they both eliminate the syndrome of patients with knee osteoarthritis. Their wavelengths combine 0.65 to 0.66 μm and 10.6 μm (Fig. 9C).

#### 3.5.4. MCID pass rate

The MCID attainment rates of WTS, PS, and stiffness score were higher in Multi L patients than in Single L patients. However, the WOMAC physical function score MCID rate of LA patients was higher than that of Multi L patients. In the comparison of Multi L versus Single L, the WTS was 35.48% in Multi L and 32.20% in Single L. The WOMAC pain score was 35.48% in Multi L and 32.20% in Single L. The WOMAC stiffness score was 61% in Multi L. The MCID rates were higher in LA patients than in Single L patients. The WOMAC stiffness score for Multi L was 61.28%, and for Single L was 32.20%. The WOMAC physical function score for Multi L was 0, and for Single L was 27.12%.

#### 3.5.5. Patient’s self-assessment

Two studies reported the PSA score outcome in multi-L compared with L.^[21,24]^ The pooling results showed non-significant multi-L results compared to the L alone (MD: 1.31 [0.97, 1.78], I^2^ = 0%), showing that the multi-L is significantly more effective than L. Their wavelengths combine 0.65 to 0.66 μm and 10.6 μm (Fig. 9D).

### 3.6. LA + muscle training versus muscle training

One trial compared the LA with the muscle training, which depicts an increment in both the experimental and the control groups regarding the LKSS score, Extensor and Flexor Peak Torque (PT), and Peak torque/weight (PT/BW) value.^[23]^ However, the amplitude in the outcomes of the experimental group (combined laser acupuncture and muscle training) was higher.^[23]^ Regarding serum biomarkers, IL-6 IL-1 was diminished, while Sox 9 and collagen II were added after treatment in both groups.^[23]^ Nevertheless, the experimental group had a higher amplitude than the control group.^[23]^ Overall, applying a laser could relieve pain and improve the function of the knee and its related muscles without any adverse events.^[23]^ The combined application of laser and muscle training has a superior effect than muscle training alone.^[23]^

### 3.7. LA versus LA + HM versus HM

One study conducted a 3-arm trial to compare the efficacy of LA plus HM versus. LA versus. HM, showing that the VAS score, tumor necrosis factor-alpha of LA combined with HM was lower than LA or HM alone, while the LKSS score is significantly higher than the other 2 groups, which indicates the combination of LA and HM could relieve knee pain symptoms, improve function as well as delay disease progression.^[25]^

### 3.8. Laser moxibustion (LM) versus M/LM versus Sham M

One study compared the LM besides the traditional moxibustion group, showing that by using LM, it could achieve the equivalent influence in most outcomes (WOMAC pain, stiffness, and physical function) at the mid-term, end of therapy, and follow-up, even has superior effect in some areas (physical function, physiological discomfort and mental wellness at the follow-up stage).^[29]^ The study compared LM with sham moxibustion, finding that, at week 4, the active group’s WOMAC pain score was significantly lower than the sham groups.^[30]^ When compared to a sham laser, active laser treatment has significantly reduced discomfort and improved function by week 24.^[30]^ When comparing the active group to the sham control group, the physical component of quality of life improved significantly between weeks 4 and 24.^[30]^ The effect of LM and M in RP, SF, and body pain were almost equivalent, but in Physical Functioning, the LM can perform even better than traditional moxibustion.^[29]^ There were no significant side effects noted.^[29]^

## 4. Discussion

**Figure 10.**
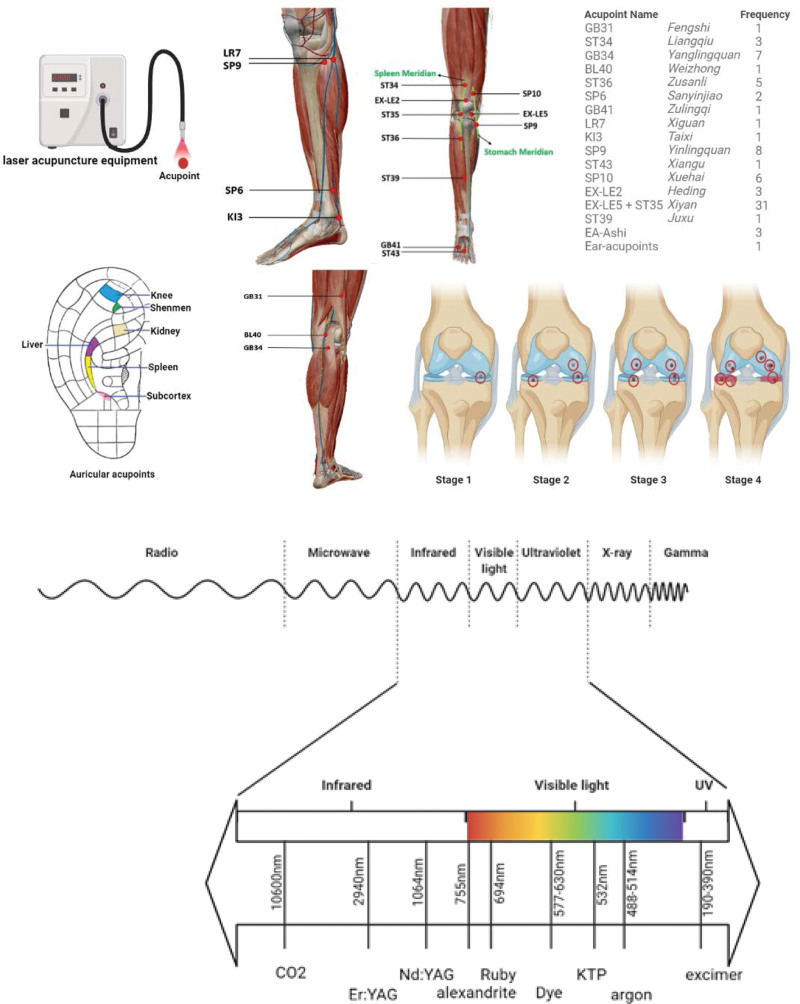
The diagram of laser acupuncture, included acupoints and used frequency, and OA 4 stages.^[26]^

Knee osteoarthritis is a debilitating condition characterized by knee pain and dysfunction, potentially leading to disability without timely intervention.^[41]^ The pain associated with OA can even disrupt static balance.^[41,42]^ Among knee disorders, rheumatoid arthritis, osteoarthritis, and osteoporosis are prevalent, with osteoarthritis and rheumatoid arthritis being the most common forms of arthritis.^[42,43]^ It is possible for an individual to have both osteoporosis and osteoarthritis concurrently.^[43]^ This study focuses solely on knee osteoarthritis, which progresses through 4 stages: minor, mild, moderate, and severe, as illustrated in Figure 10.^[44]^ Patients in Stage 1 of OA exhibit modest wear, tear, and bone spur growth at the knee joint ends without experiencing pain.^[44,45]^ Lifestyle modifications can be beneficial at this stage.^[44,45]^ Stage 2 is characterized by increased bone spur formation, joint pain, stiffness, and discomfort, with proteolysis breaking down the cartilage matrix due to enzyme production like metalloproteinases, despite the cartilage and soft tissues remaining healthy.^[44,45]^ Exercise and strength training regimens can help alleviate pain.^[44,45]^ In Stage 3, erosion of the cartilage surface and bone gap narrowing due to fibrillation occur, leading to bone spur formation from proteoglycan and collagen fragments.^[44,45]^ Nonsteroidal anti-inflammatory drugs (NSAIDs), codeine, and oxycodone are effective treatments.^[46,47]^ Stage 4 is characterized by full-thickness cartilage lesions, bone marrow edema patterns, and chronic inflammatory responses triggered by cartilage destruction, resulting in the formation of new spurs and severe pain.^[46,47]^ Stage 4 typically requires knee replacement or osteotomy for management.^[46,47]^

Acupuncture, a fundamental element of TCM, involves the flow of qi in acupoints and meridians, with acupoint selection being crucial for effective treatment.^[48,49]^ For the acupoints used in treating knee OA, scientists have concluded that *Shangqiu* (SP5), *Qiuxu* (GB40), and *Jiexi* (ST41) are the most helpful distal acupoints, *Liangqiu* (ST34) is mainly for pain above the knee, *Zusanli* (ST36), *Yanglinquan* (GB34), and GB33 are central in pain on the lateral side, *Yinlingquan* (SP9), *Xiguan* (LR7), and *Ququan* (LR8) are beneficial for, pain on the inner side.^[50]^ *Zulingqi* (GB41) and *Waiguan* (TB5) help open the Yang Linking Vessel, commonly used for the lateral pain of knees.^[50]^ At the same time, *Gongsun* (SP4) and *Neiguan* (PC6) are crucial in opening the penetrating vessel, helping relieve the medial pain of the knees.^[28]^ We concluded that acupoints and their frequencies were used in 25 included studies in Figure 10.

Nevertheless, adverse reactions may thread the widespread development of acupuncture, with the most common cases being fainting during treatment, pneumothorax, local skin infection, nausea, vomiting, perichondritis and increased pain, while some rare cases are burns from moxa, nerve injury, forgotten needle, menstruation disturbance, chylothorax, lymphedema, and insomnia.^[51,52]^ Recently, Xu et al also did a systematic review of 117 reports (including a total of 308 adverse events), aiming to conclude the adverse events of acupuncture, finding that bacterial infection, especially mycobacterial and staphylococcal, was the main complication after acupuncture.^[53]^ Besides, some reusable needles may also cause hepatitis, bruising, bleeding, dizziness, fainting, puncture of organs, etc, due to improper insertion.^[53]^ The moxibustion could produce around 10 µm infrared radiation spectra into the acupoints whose peak wavelength is 10 µm that is located in the human body, hence initiating resonance absorption, increasing temperature, in which heat-sensitive immune cells, heat-shock proteins, and certain receptors are activated via bodily fluid and neural routes, resulting in stimulus signals and consequences that are then sent to distant organs and the entire body, hence achieving a better efficacy of treatment.^[49]^ However, traditional moxibustion may produce undesirable smoke and smell.^[54]^ Besides, some researchers suggest that the allergy may occur in the patients after moxibustion treatment.^[54]^

Notably, laser acupuncture, compared to traditional methods like MA, electro-acupuncture, and moxibustion, offers a noninvasive approach with minimal side effects and no smoke production.^[55]^ Laser therapy, particularly low-level laser therapy, has shown promise in managing chronic low back pain, with specific parameters like wavelength and frequency influencing treatment outcomes.^[55]^

Diode laser emitting or carbon dioxide (CO_2_) laser emitting is widely used in LA apparatus.^[56]^ A diode laser is a beam transmitted through a sturdy fiber-optic cable with a quartz core that runs from the base unit to the operating location.^[56]^ There are 2 modes available for the diode laser, consisting of noncontact mode (more diffuse with deeper penetration) and contact mode (more accurate and controlled).^[57]^ Scientists also tend to combine a visible light beam with an invisible laser beam with various types of shapes, including flat, conical, orb tips, etc, aimed for better control targeting.^[58]^ The wavelength emitted from the laser beam is determined by the blasting media, which owns various types, ranging from 600 to 1300 nm (e.g., gallium arsenide [GaAs, 904 nm], helium–neon [HeNe, 632.8 nm], and gallium–aluminum–arsenide [GaAlAs, 820 nm]).^[59]^ CO_2_ laser is often used in the included studies after screening literature.^[14–38]^ CO2 laser acupuncture serves as a heat source for spot moxibustion, being an invisible infrared laser, with its raw laser power as the most substantial benefit.^[60,61]^ Although the CO_2_ laser has higher power, it is massive, bulky, and has relatively fragile mirror systems, making it hard to transport and place.^[11,62,63]^ In contrast with the CO_2_ fiber, a diode fiber needing repair can be curtailed and reutilized, which is better for post-maintenance and long-term use.^[60]^ Neon (He-Ne) laser has a specific stimulating effect on acupuncture points of the human body, and it can fully reach the depth of 10mm~15mm of the tissue.^[50]^ A krypton ion (Kr) laser can be used for intense stimulation therapy.^[53]^ CO_2_ laser irradiation has thermal and stimulating effects on acupoints, but does not penetrate deep into the tissue (about only 0.21 mm), which could enable it to act on superficial layers of the skin.^[61]^ The neodymium-doped yttrium aluminum garnet laser could be used if deep tissues need a strong stimulating effect.^[64]^ The dose of laser irradiation must be controlled well to prevent undesirable damage.^[65]^ We concluded the most used laser types and spectrum in Figure 10.

LA involves thermal energy (photothermal energy), electrical energy, and mechanical energy.^[66]^ Under the action of an electromagnetic wave in the field, positive and negative ions in the inner and outer fluids of the cells move in opposite directions, generating action potentials and electrical signals and stimulating the receptors at the acupoints.^[67]^

Laser can affect the permeability of cell membranes and the activity of some enzymes and speed up the operation of nutrients by improving blood circulation, thus strengthening the body’s metabolism and improving the body’s functions.^[57,58]^ Laser irradiation increases the collagen production of fibroblasts and accelerates the reproduction of angiogenic cells, it also can promote the ossification of the fracture site, and the local vascular network can become more abundant in bone columnar structure.^[57,58]^ Low light levels can activate cells by dividing, migrating, and altering their metabolism, especially if the light is flashed on and off.^[11,68]^ Light therapy that can be pulsed on and off would be better as not to heat the tissues.^[11,68]^ Apart from bulky medical instruments, the above 3 qualities can be found in some portable devices such as *Power Me* of *Raymedy* (https://www.raymedy.hk/), which could achieve a more convenient and real-time treatment.

The power density plays a crucial role in getting the proper effect for patients with knee OA.^[33]^ Dwi R. Helianthi mentioned that lower laser doses may contribute to different efficacy of treatment.^[33]^ Besides, parameters like laser wavelength, output power, energy density, and duration of treatment would also influence the laser doses.^[33,57,58,62,63]^ In this study, we analyzed the efficacy and safety of laser acupuncture on patients with knee osteoarthritis based on different study design classifications. However, there needs to be more detailed information about their laser parameters. Meanwhile, the outcomes are primarily heterogeneous because of their unrigorous study conductance, which may lead to a higher risk of bias, especially a high risk of allocation concealment and blindness. A few included studies described in more detail whether the lesions were located in the left or right leg joint as the effect is different from that on the opposite side of the affected limb. On top of that, the season and time-point should be considered in the trials of included studies, which is the limitation of current included studies. The relatively small sample size and short treatment duration are another drawback. More studies are expected to have a solid conclusion in the future.

## 5. Conclusion

The meta-analysis presented here highlights the potential superiority of utilizing Laser Acupuncture in patients with Osteoarthritis (OA), particularly when employing specific laser parameters (810 nm, 785 nm). Comparing LA to traditional acupuncture, it shows comparable effectiveness in pain relief and morning stiffness alleviation. Moreover, the study suggests that stimulating more acupoints during laser acupuncture treatment enhances its efficacy. Research indicates that employing multiple acupoints leads to improved clinical outcomes in terms of WOMAC total and physical function scores. Combining laser acupuncture with isokinetic muscle training proves more beneficial for knee OA patients compared to muscle training alone.

Numerous articles yield varied results due to inconsistent disease staging in patient research. Furthermore, the selection of acupoints lacks scientific rigor, with remote acupoints often not correlating with those commonly used. Some acupoints are chosen without sufficient evidence, emphasizing the importance of adhering to the principles in TCM theory when selecting acupoints. While some studies focus on single acupoints, TCM emphasizes the interconnectedness of acupoints, suggesting that they should not be treated in isolation for optimal results. Many studies fail to progress from a macro to micro approach in LA, neglecting the exploration of acupoints and meridians before delving into their molecular mechanisms. The establishment of parameters, such as light frequency, lacks scientific basis, with output power often not adjusted according to symptom relief progression. Variability in laser parameters across studies makes outcome comparison challenging. Standardizing disease severity, parameters, and outcomes is essential for future research to consolidate and build upon these findings.

## Acknowledgments

We want to pay our authentic thankfulness to the funding support of Raymedy Bio-Energy InnoTech Limited. I also wish to acknowledge the support of the City University of Hong Kong studentship.

## Author contributions

**Conceptualization:** Rong Han, Jinlian Hu.

**Data curation:** Rong Han, Chunxia Guo.

**Formal analysis:** Rong Han, Chunxia Guo.

**Funding acquisition:** Jinlian Hu.

**Investigation:** Rong Han, Chunxia Guo, Kit Lau, Jinlian Hu.

**Methodology:** Rong Han, Jinlian Hu.

**Project administration:** Rong Han, Chunxia Guo, Kit Lau, Jinlian Hu.

**Resources:** Rong Han, Jinlian Hu.

**Software:** Rong Han.

**Supervision:** Jinlian Hu.

**Validation:** Rong Han, Chunxia Guo.

**Visualization:** Rong Han, Chunxia Guo.

**Writing – original draft:** Rong Han.

**Writing – review & editing:** Rong Han, Chunxia Guo, Kit Lau, Jinlian Hu.
